# Temperature regulation of carotenoid accumulation in the petals of sweet osmanthus via modulating expression of carotenoid biosynthesis and degradation genes

**DOI:** 10.1186/s12864-022-08643-0

**Published:** 2022-06-04

**Authors:** Yiguang Wang, Chao Zhang, Bin Xu, Jianxin Fu, Yanxia Du, Qiu Fang, Bin Dong, Hongbo Zhao

**Affiliations:** Zhejiang Provincial Key Laboratory of Germplasm Innovation and Utilization for Garden Plants, Key Laboratory of National Forestry and Grassland Administration on Germplasm Innovation and Utilization for Southern Garden Plants, Department of Ornamental Horticulture, School of Landscape Architecture, Zhejiang Agriculture and Forestry University, 311300 Hangzhou, China

**Keywords:** Sweet osmanthus, Floral color, Carotenoids, Temperature, Gene expression

## Abstract

**Background:**

Temperature is involved in the regulation of carotenoid accumulation in many plants. The floral color of sweet osmanthus (*Osmanthus fragrans* Lour.) which is mainly contributed by carotenoid content, is affected by temperature in autumn. However, the mechanism remains unknown. Here, to reveal how temperature regulates the floral color of sweet osmanthus, potted sweet osmanthus ‘Jinqiu Gui’ were treated by different temperatures (15 °C, 19 °C or 32 °C). The floral color, carotenoid content, and the expression level of carotenoid-related genes in petals of sweet osmanthus ‘Jinqiu Gui’ under different temperature treatments were investigated.

**Results:**

Compared to the control (19 °C), high temperature (32 °C) changed the floral color from yellow to yellowish-white with higher lightness (L*) value and lower redness (a*) value, while low temperature (15 °C) turned the floral color from yellow to pale orange with decreased L* value and increased a* value. Total carotenoid content and the content of individual carotenoids (α-carotene, β-carotene, α-cryptoxanthin, β-cryptoxanthin, lutein and zeaxanthin) were inhibited by high temperature, but were enhanced by low temperature. Lower carotenoid accumulation under high temperature was probably attributed to transcriptional down-regulation of the biosynthesis gene *OfPSY1*, *OfZ-ISO1* and *OfLCYB1*, and up-regulation of degradation genes *OfNCED3*, *OfCCD1-1*, *OfCCD1-2*, and *OfCCD4-1*. Up-regulation of *OfLCYB1*, and down-regulation of *OfNCED3* and *OfCCD4-1* were predicted to be involved in low-temperature-regulated carotenoid accumulation. Luciferase assays showed that the promoter activity of *OfLCYB1* was activated by low temperature, and repressed by high temperature. However, the promoter activity of *OfCCD4-1* was repressed by low temperature, and activated by high temperature.

**Conclusions:**

Our study revealed that high temperature suppressed the floral coloration by repressing the expression of carotenoid biosynthesis genes, and activating the expression of carotenoid degradation genes. However, the relative low temperature had opposite effects on floral coloration and carotenoid biosynthesis in sweet osmanthus. These results will help reveal the regulatory mechanism of temperature on carotenoid accumulation in the petals of sweet osmanthus.

**Supplementary Information:**

The online version contains supplementary material available at 10.1186/s12864-022-08643-0.

## Introduction

Sweet osmanthus (*Osmanthus fragrans* Lour.), a member of Oleaceae family, is valued as an ornamental tree for its vivid floral color and sweet floral aroma [1]. The flowers of sweet osmanthus also have high commercial values as a flavor additive for food or drinks [2]. According to the various floral color, sweet osmanthus cultivars were divided into three groups, including Albus group with white, greenish or yellowish white floral color, Luteus group showing yellow or golden floral color, and Aurantiacus group with range of floral color from pale orange to orange red [1]. Carotenoids are the major pigments determining the floral color variation of sweet osmanthus cultivars [3, 4]. Aside from providing color traits for flowers or fruits of many plants [5, 6], carotenoids which are precursors for synthesis of vitamin A and antioxidants for resistance to disease, are beneficial for human health [7, 8]. The carotenoid components which have been identified in flowers of 24 sweet osmanthus cultivars, include α-carotene, β-carotene, α-cryptoxanthin, β-cryptoxanthin, lutein, and zeaxanthin [4]. Among these carotenoids, β-carotene is the most dominate one in both yellowish-white and orange-red clusters of sweet osmanthus cultivars [4].

The carotenoid metabolism in plants consists of biosynthesis and degradation pathway, involving multiple enzymes [9]. In carotenoid biosynthesis pathway, enzymes like phytoene synthase (PSY), phytoene desaturase (PDS), ζ-carotene isomerase (Z-ISO), ζ-carotene desaturase (ZDS), and carotenoid isomerase (CRTISO) contribute to the synthesis of a red carotene, lycopene. Subsequently, lycopene is cyclized into α-carotene by the function of ε-ring cyclase (LCYE) and β-ring cyclase (LCYB), or cyclized into β-carotene with catalysis of LCYB. These two carotenes are respectively catalyzed to produce various xanthophylls by enzymes such as ε-ring hydroxylase (CHYE), β-ring hydroxylase (CHYB), zeaxanthin epoxidase (ZEP), violaxanthin de-epoxidase (VDE) and neoxanthin synthase (NSY). In carotenoid degradation pathway, carotenoid cleavage dioxygenases (CCDs) or 9-*cis*-epoxycarotenoid dioxygenases (NCEDs) cleave carotenoids to produce apocarotenoids or the ABA precursor. The key enzymes affecting change of floral color depend on species, such as PSY in golden-orange and white flowers of california poppy (*Eschscholzia californica*) [10], CHYB, ZEP and CCD1 in *Oncidium* cultivars [11], CHYB in yellow petals of *Ipomoea* sp. and white petals of *I. nil* and *I. obscura* [12]. Previous studies suggested that differential expression of *OfCCD4* is the key reason for the diversity in the total carotenoid concentrations in yellowish-white and orange-red clusters of sweet osmanthus cultivars [3, 4].

Most cultivars of sweet osmanthus are blooming in autumn (from the end of August to November) in China [1]. During that period, temperature fluctuates greatly, which may cause changes in floral color of sweet osmanthus between different years. For example, the floral color of cultivars in Albus group and Luteus group turned darker during cold years or months, or the floral color of cultivars in Aurantiacus group turned paler during warm years or months. In our previous study on the effect of temperature on the floral scent production in sweet osmanthus [13], it is easy to notice this phenomenon in different temperature treatments with naked eyes. Therefore, it remains to confirm whether high or low temperature regulated carotenoid accumulation in the flowers of sweet osmanthus, resulting in the change of floral color.

Temperature indeed plays important roles in regulating carotenoid metabolism in many horticulture plants, such as tomato (*Solanum lycopersicum*) [14–16], banana (*Musa acuminata*) [17], Satsuma mandarin (*Citrus unshiu*) [18], navel orange (*Citrus sinensis*) [19], papaya fruit (*Carica papaya*) [20], and albino tea (*Camellia sinensis*) [21]. High temperature can strongly reduce biosynthesis and accumulation of lycopene in tomato fruits [22]. Similarly, 30 °C of storage temperature decreased the content of carotenoid in juice sacs of Satsuma mandarin, accompanying with down-regulation of most detected carotenoid biosynthesis-related genes [18]. As to the effect of low temperature on carotenoid biosynthesis, two regions of low temperature need to be considered: relative low temperature and chilling temperature (< 10 °C). With the decrease of temperature from 30 °C, lycopene gradually decreased in tomato fruits, stayed the same at a broader plateau (18–26 °C) and was sharply reduced at 14 °C [16], suggesting that carotenoid biosynthesis would be inhibited by low temperature when it is lower than a certain extent. However, the regulation of low temperature on carotenoid content is quite different in different tissues or cultivars. For instance, the carotenoid accumulation showed different trend in two banana cultivars under the same cold storage condition [17]. Compared to 20 °C, storage at 4 °C induced carotenoid accumulation in pulp of navel orange ‘Cara Cara’, whereas reduced carotenoid content in peel [19].

Researches about the regulation of temperature on carotenoid metabolism are mainly focused on fruits and vegetables, and however, much fewer researches were reported on the influence of temperature on the carotenoids profile in ornamental plants. In an epiphytic orchid, *Psygmorchis pusilla*, 32 °C results in low levels of carotenoids [23]. But temperature doesn’t affect the total amount of carotenoid in spray chrysanthemum [24]. At present, similar study has not been reported in sweet osmanthus. Thus, this study used potted sweet osmanthus ‘Jinqiu Gui’ (a cultivar in Luteus group) to find out the effects of high and low temperature on the floral color, the content of carotenoids, and expression of carotenoid-related genes in petals during flower opening. Furthermore, the promoter activities of key genes in response to different temperatures were investigated. Results of the present study will lay a theoretical foundation for revealing the regulation mechanism of temperature on carotenoid biosynthesis and floral color, and help develop postharvest techniques for high-content carotenoids in the petals of sweet osmanthus.

## Results

### Effects of different temperatures on floral color in sweet osmanthus

**Fig. 1:**
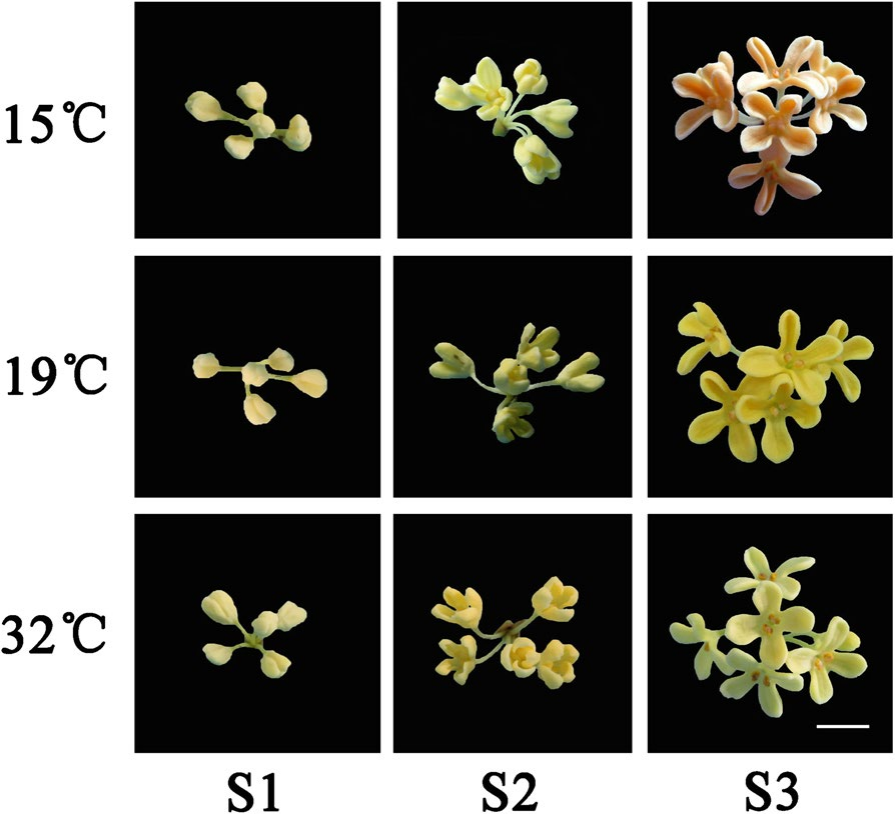
Floral color of sweet osmanthus ‘Jinqiu Gui’ with treatments of different temperatures (15 °C, 19 °C or 32 °C). S1: Linggeng stage; S2: Initial flowering stage; S3: Full flowering stage. Bar: 5 mm

As Fig. 1 shown, each treatment was divided into three flowering stages, including linggeng stage (S1, the inflorescence burst through bracts and the florets closely crowded), initial flowering stage (S2) and full flowering stage (S3). During the first two flowering stages, all floral colors of three treatments were similar, showing pale lemon-yellow or lemon-yellow with naked eye observation. When flowers fully opened, floral colors of three treatments were extremely different. At S3, compared to the yellow flowers of ‘Jinqiu Gui’ in the control treatment (19 °C), high temperature (32 °C) changed the floral color into yellowish-white. However, the floral color in low temperature (15 °C) was visibly darker than that of control, showing pale orange.

**Fig. 2:**
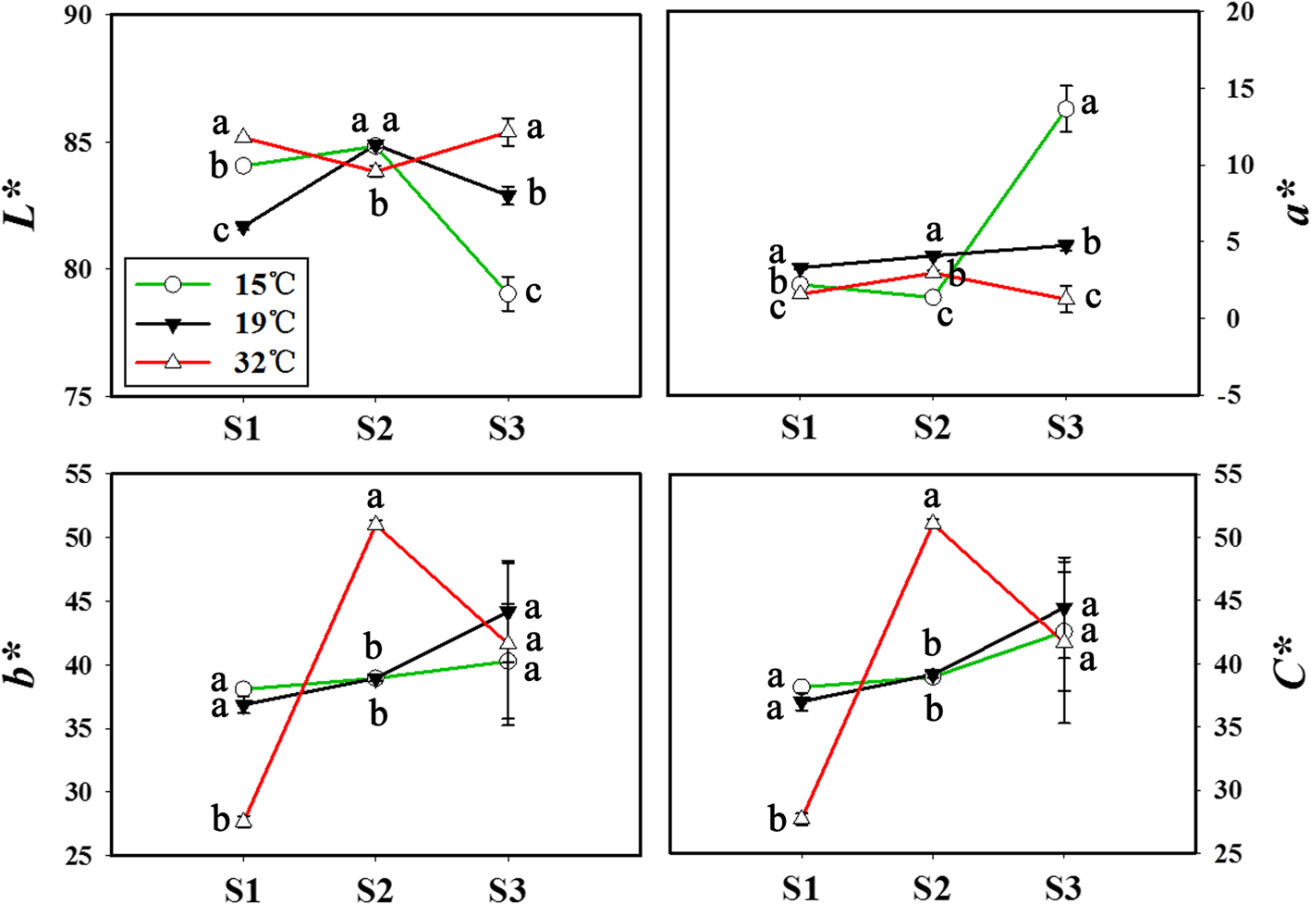
Changes of floral color parameters of sweet osmanthus ‘Jinqiu Gui’ with treatments of different temperatures. Floral color parameters include L*, a*, b* and C*. S1: Linggeng stage; S2: Initial flowering stage; S3: Full flowering stage. Values with the different letters are statistically different according to Duncan’s multiple range test at *P* < 0.05

In order to obtain quantifiable results of floral color, colorimeter was used to measure chromatic components. Changes of floral color parameters lightness (L*), redness (a*), yellowness (b*) and chroma (C*) with treatments of different temperatures are showed in Fig. 2. Compared to the floral color parameters of the control treatment at S3, high temperature greatly increased L* value, and reduced a* value. However, low temperature significantly reduced L* value, and increased a* value. Either of high temperature and low temperature had no effect on b* and C* values at S3.

### Effects of different temperatures on carotenoid content in sweet osmanthus

**Fig. 3:**
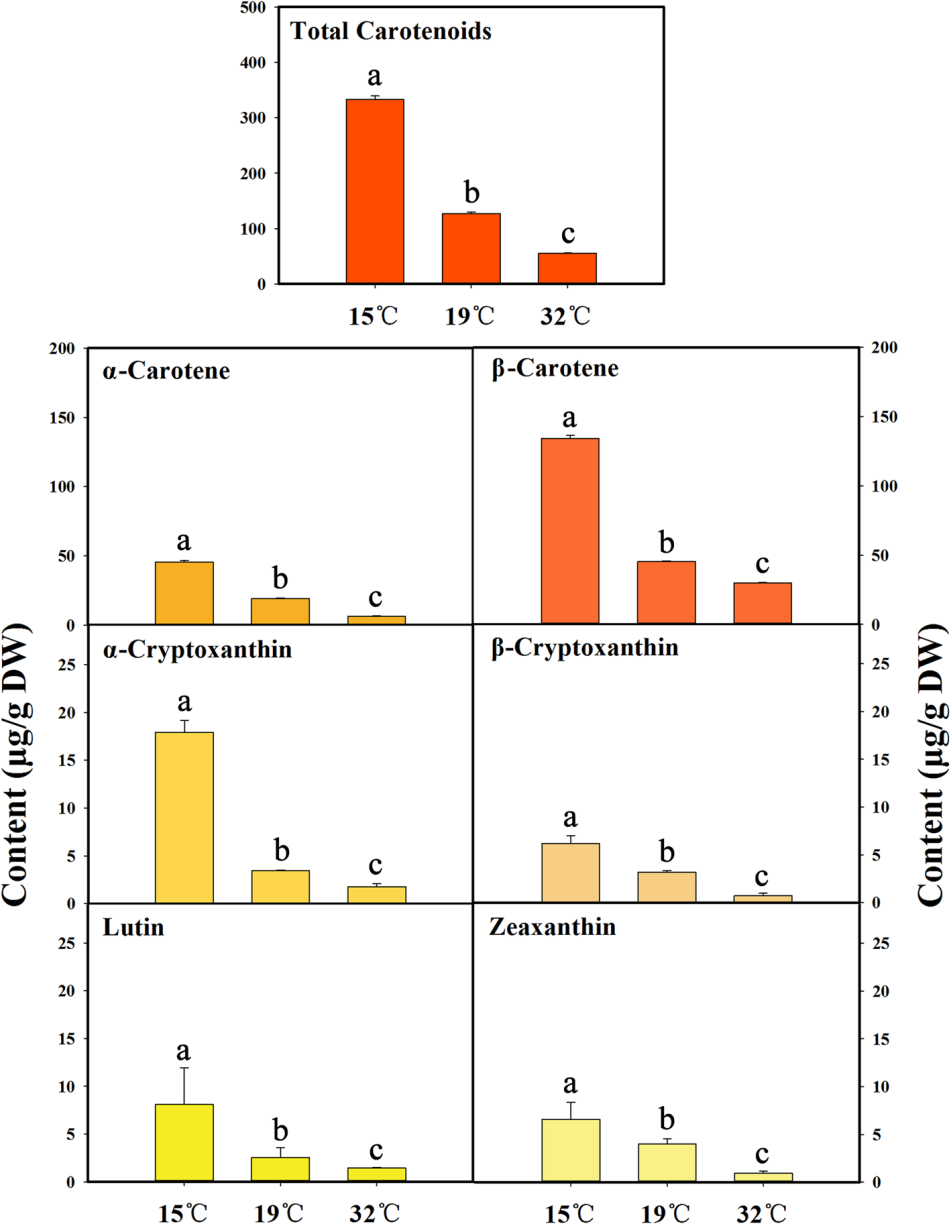
Content of carotenoids in petals of sweet osmanthus ‘Jinqiu Gui’ at full flowering stage with treatments of different temperatures. Values with the different letters are statistically different according to Duncan’s multiple range test at *P* < 0.05

The carotenoid components in petals of sweet osmanthus (Fig. S1) were identified according to our previous study [4]. Figure 3 shows total carotenoid content and the content of individual carotenoid components, including α-carotene, β-carotene, α-cryptoxanthin, β-cryptoxanthin, lutein, and zeaxanthin in petals of sweet osmanthus with different temperature treatments. Compared to total carotenoids in the control, total carotenoids in high temperature (32 °C) decreased by 56.55%. Specifically, the content of six carotenoids (α-carotene, β-carotene, α-cryptoxanthin, β-cryptoxanthin, lutein and zeaxanthin) declined under high temperature condition. On the contrary, low temperature (15 °C) increased total carotenoid content by 161.74% in petals of sweet osmanthus. The content of each carotenoid (α-carotene, β-carotene, α-cryptoxanthin, β-cryptoxanthin, lutein and zeaxanthin) also increased with low temperature treatment.

### Effects of different temperatures on the expression levels of carotenoid-related genes

**Fig. 4:**
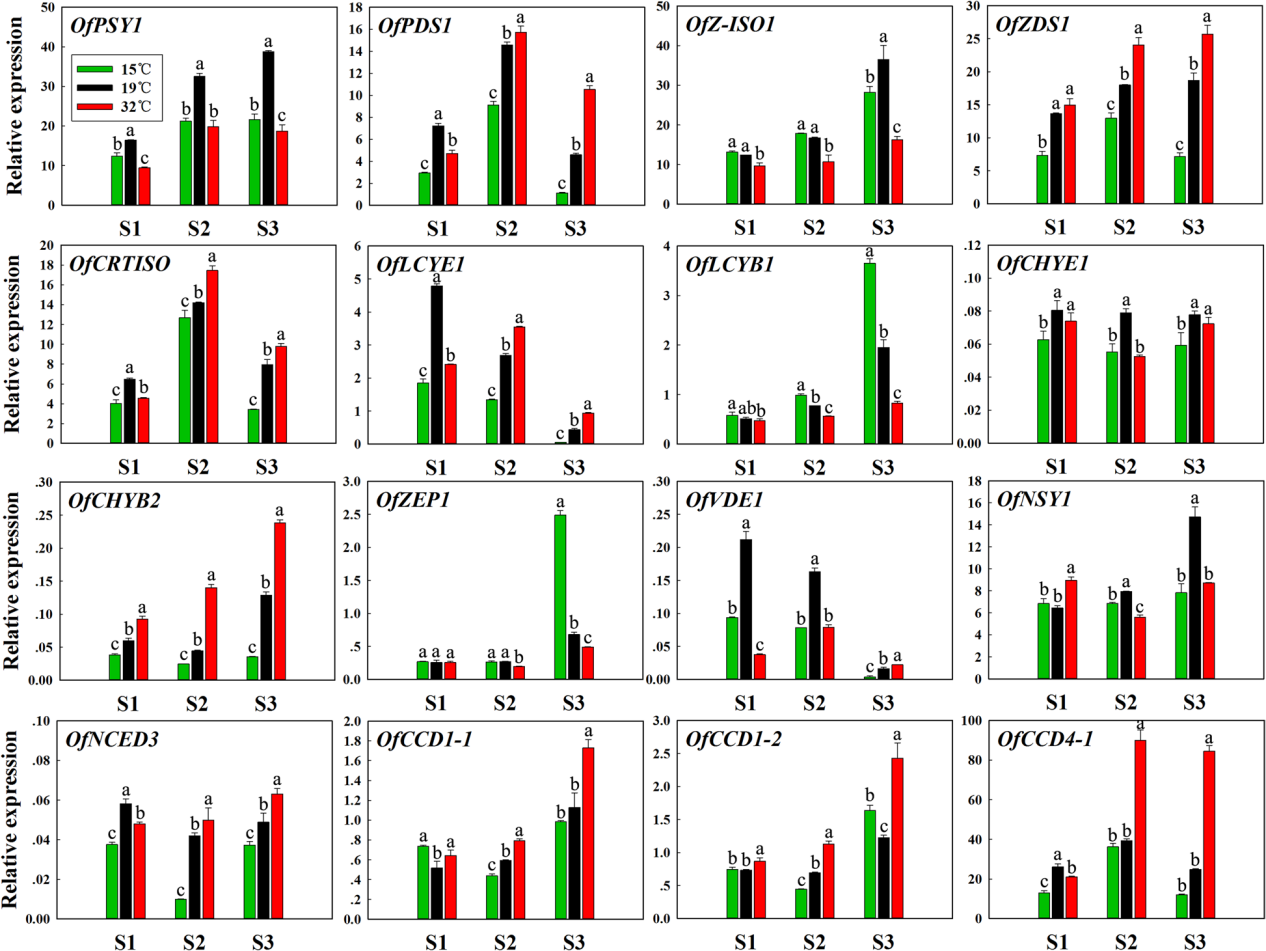
The expression levels of carotenoid-related genes in petals of sweet osmanthus ‘Jinqiu Gui’ under different temperature treatments. S1: Linggeng stage; S2: Initial flowering stage; S3: Full flowering stage. Values with the different letters are statistically different according to Duncan’s multiple range test at *P* < 0.05

Because the carotenoid content in petals of sweet osmanthus ‘Jinqiu Gui’ changed with different temperature treatments, the expression levels of related genes were detected as well (Fig. 4). The results show that the expression patterns of some carotenoid biosynthesis and degradation genes correlated with the carotenoid accumulation. Compared to the control, high temperature treatment repressed the expression levels of genes in carotene biosynthesis, like *OfPSY1* and *OfZ-ISO1* from S1 to S2, *OfLCYB1* from S2 to S3. However, with high temperature treatment, the xanthophylls biosynthesis gene *OfCHYB2*, degradation genes *OfCCD1-1* and *OfCCD1-2* were up-regulated at three flowering stages. Degradation genes *OfNCED3* and *OfCCD4-1* were up-regulated at S2 and S3. The other genes in xanthophylls biosynthesis, such as *OfZEP1*, *OfVDE1*, and *OfNSY1* were down-regulated at two or three stages by high temperature.

With low temperature treatment, many genes in carotene biosynthesis, including *OfPSY1*, *OfPDS1*, *OfZDS1*, *OfCRISO1* and *OfLCYE1* were down-regulated in petals of ‘Jinqiu Gui’ during three flowering stages. However, the expression levels of *OfLCYB1* were up-regulated in petals of ‘Jinqiu Gui’ at S2 and S3. Although the expression levels of *OfZEP1* were up-regulated at S3, the other xanthophylls biosynthesis genes, like *OfCHYE1*, *OfCHYB2*, *OfVDE1*, and *OfNSY1* were down-regulated at two or three stages by low temperature. The expression levels of degradation genes *OfNCED3* at three stages, *OfCCD4-1* at two stages, *OfCCD1-1* and *OfCCD1-2* at S2 were down-regulated.

### *Cis*-acting elements in promoters of* OfLCYB1* and *OfCCD4-1*

**Fig. 5:**
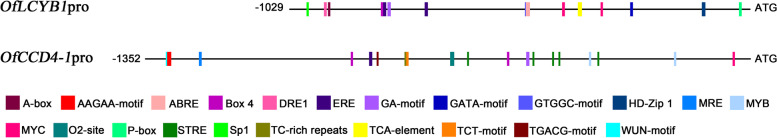
*Cis*-acting elements in promoters of *OfLCYB1* and *OfCCD4-1*

Among these tested genes, *OfLCYB1* is directly responsible for production of β-carotene, which is the most dominate carotenoid compound in petals of sweet osmanthus, and *OfCCD4-1* is the key degradation gene for carotenoids in sweet osmanthus in this study and our previous study [4]. Therefore, 1029 bp promoter of *OfLCYB1* and 1352 bp promoter of *OfCCD4-1* were cloned from genomic DNA of ‘Jinqiu Gui’, and used for *cis*-acting element analysis by using online website PlantCARE. As Fig. 5 shown, in *OfLCYB1* promoter, there are *cis*-acting elements being involved in light responsiveness (such as Sp1, Box 4, GA-motif, GTGGC-motif, GATA-motif), a dehydration-responsive element (DRE1), an A-box, two ethylene-responsive elements (ERE), an ABA-responsive element (ABRE), two MYC binding sites, a TCA-element involving in salicylic acid responsiveness, an HD-Zip 1 element being involved in differentiation of the palisade mesophyll cells, a gibberellin-responsive element (P-box), etc. In *OfCCD4-1* promoter, there are a wound-responsive element (WUN-motif), an AAGAA-motif, three MYB binding sites, several light-responsive elements (Box 4, TCT-motif, GA-motif), an ethylene-responsive element (ERE), an MeJA-responsive element (TGACG-motif), five stress responsive elements (STRE), a TC-rich repeats involved in defense and stress responsiveness, an O2-site being involved in zein metabolism regulation, and an MYC site, etc.

### Effects of different temperatures on the promoters of *OfLCYB1* and *OfCCD4-1*

**Fig. 6:**
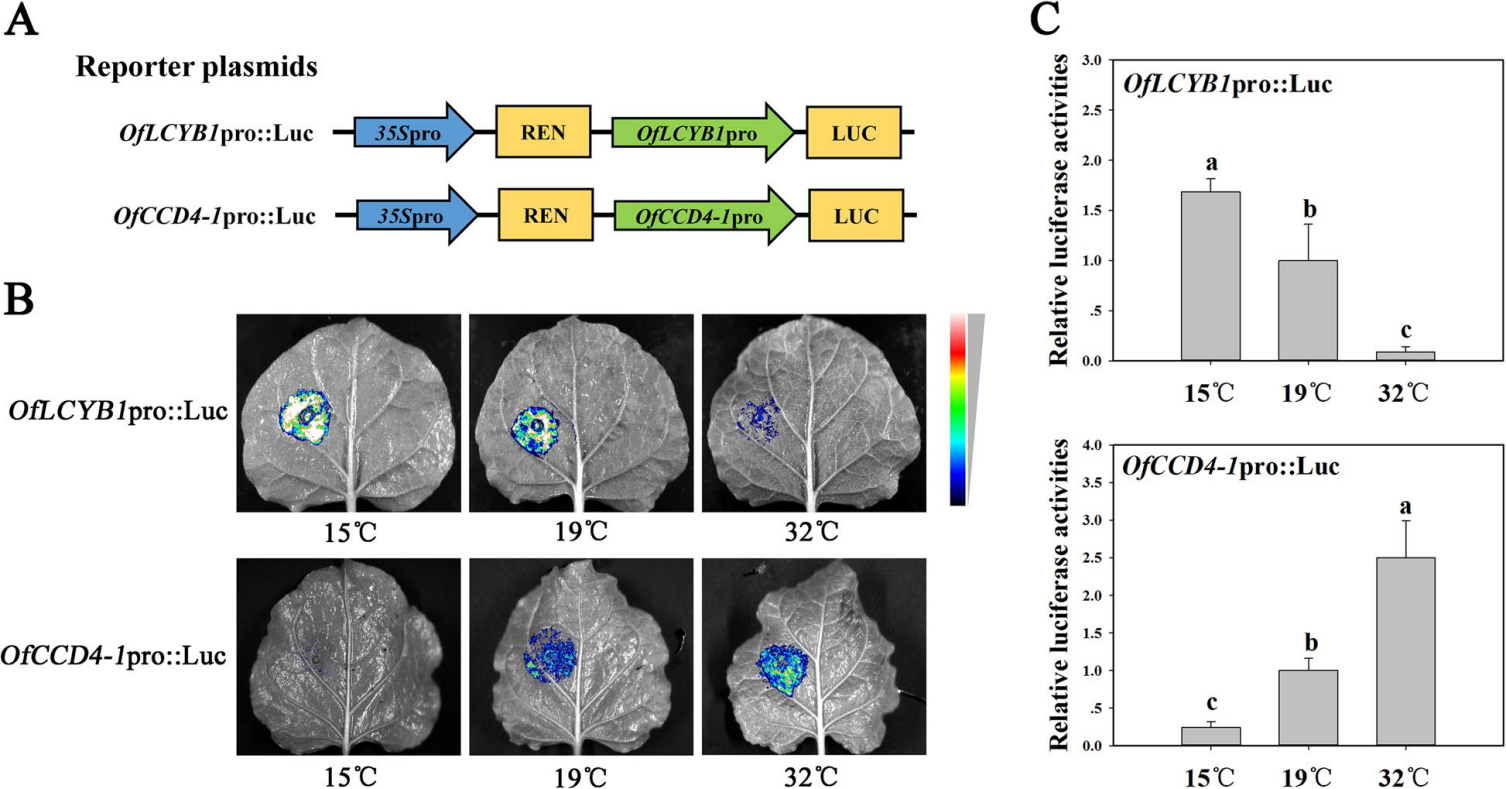
Changes in activities of *OfLCYB1* and *OfCCD4-1* promoters under different temperature treatments. **A** The pGreenII 0800-Luc constructs containing *OfLCYB1* promoter or *OfCCD4-1* promoter were used as reporter plasmids for luciferase assays. **B** Luciferase assays by transiently transformation into in *Nicotiana benthamiana* leaves with *OfLCYB1*pro::Luc and *OfCCD4-1*pro::Luc under 15 °C, 19 °C or 32 °C. The bar showing blue to red indicates luciferase signal intensity from low to high. **C** Quantitative analysis of luciferase signal intensity in different temperature treatments. The value of each control (19 °C) was set as 1. Values with the different letters are statistically different according to Duncan’s multiple range test at *P* < 0.05

The promoter sequences of *OfLCYB1* and *OfCCD4-1* were inserted into a pGreenII 0800-Luc vector, respectively (Fig. 6A). Transient luciferase assays were performed to investigate the regulation of *OfLCYB1* and *OfCCD4-1* by temperature. Figure 6B and C show that while the temperature increased from 15 °C, 19 °C to 32 °C, the luciferase signal intensity of *OfLCYB1*pro::Luc decreased. However, compared to the control (19 °C), the signal intensity of *OfCCD4-1*pro::Luc was lower in 15 °C, and higher in 32 °C. These results indicate that the promoter activity of *OfLCYB1* is activated by low temperature, and repressed by high temperature. The promoter activity of *OfCCD4-1* is repressed by low temperature, and activated by high temperature.

## Discussion

Floral color is an important ornamental trait for classification of sweet osmanthus cultivars [1]. However, the floral coloration of sweet osmanthus could be affected by environment, which remains to be investigated. Here, we found that high temperature induced ‘Jinqiu Gui’ of Luteus group showing floral color of yellowish-white, the phenotype of Albus group. The floral color of ‘Jinqiu Gui’ under low temperature was pale orange, which is similar to the phenotype of Aurantiacus group. Previous studies have showed that floral color of sweet osmanthus is predominantly determined by the carotenoid content and composition [3, 4]. Carotenoid accumulation correlates to L* and a* values which are main parameters for distinguishing the floral color of different sweet osmanthus cultivars [4]. In present study, L* and a* values of floral color, and carotenoid content were significantly affected by temperatures. The content of six detected carotenoids (α-carotene, β-carotene, α-cryptoxanthin, β-cryptoxanthin, lutein and zeaxanthin) and the total carotenoid content in petals of sweet osmanthus were significantly lower in 32 °C treatment and higher in 15 °C treatment than those in 19 °C treatment. Thus, changes in floral color of sweet osmanthus under different temperature conditions are attributed to temperature-regulated carotenoid accumulation.

**Fig. 7:**
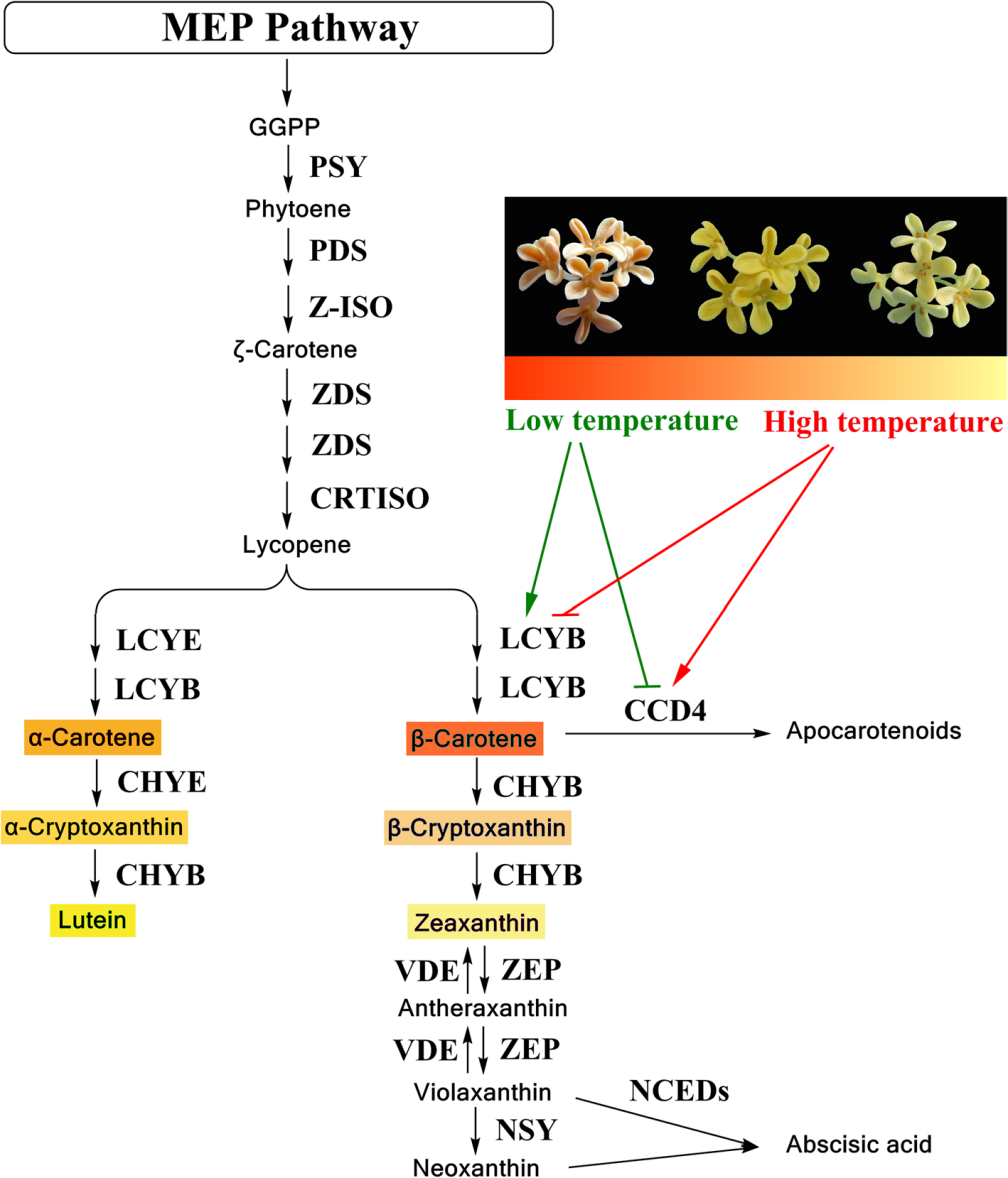
Regulation of floral coloration and carotenoid metabolic pathway in petals of sweet osmanthus by low and high temperature. The colored carotenoids (including α-carotene, β-carotene, α-cryptoxanthin, β-cryptoxanthin, lutein and zeaxanthin) in the pathway are main components in petals of sweet osmanthus

Carotenoid accumulation is determined by transcriptional regulation of carotenoid biosynthesis and degradation genes [25]. In some other plants, higher temperature also greatly reduces or inhibits carotenoid biosynthesis and accumulation [18, 22, 23]. High temperature-induced repression of carotenoid content in Satsuma mandarin is associated with lower expression levels of several carotenoid biosynthesis genes, such as *PSY*, *PDS*, *ZDS* and *LCYb* [18]. As shown in carotenoid metabolic pathway of sweet osmanthus ‘Jinqiu Gui’ (Fig. 7), decreased synthesis of α-carotene and β-carotene in petals under high temperature was probably due to down-regulation of biosynthesis genes *OfPSY1*, *OfZ-ISO1* and *OfLCYB1*. The up-regulation of *OfCHYB2* promoted the flux into down-stream products, while down-regulation of *OfVDE1* prevented converting antheraxanthin and violaxanthin back into zeaxanthin. Besides, the up-regulation of degradation genes *OfNCED3*, *OfCCD1-1*, *OfCCD1-2* and *OfCCD4-1* probably reduced total carotenoids as well as individual components. Therefore, both biosynthesis and degradation genes were regulated by high temperature, resulting in reduced carotenoid accumulation in petals of sweet osmanthus.

The effect of low temperature on carotenoid accumulation was quite complicated in other plants [17–19]. In ‘Cara Cara’ navel orange, compared with 20 °C, low temperature (4 °C) decreased the total carotenoid content in the peel, but increased the total carotenoid content in the pulp [19]. What’s more, the gene expression profiles were greatly correlated with carotenoid content in a tissue-dependent manner [19]. However, the expression of carotenogenic genes was not well correlated with carotenoid content in the flavedo and juice sacs of Satsuma mandarin fruit under low storage temperature [18]. Similarly, in this study, the response of most carotenoid-related gene expression and carotenoid accumulation to low temperature was also not well correlated. Many carotenoid biosynthesis genes were down-regulated under low temperature, but the expression level of *OfLCYB1* was much higher in the petals with 15 °C treatment, revealing that *OfLCYB1* probably plays important role in the massive accumulation of total carotenoid and individual carotenoids under low temperature. Besides, the down-regulation of xanthophylls biosynthesis genes *OfCHYE1*, *OfCHYB2* and *OfNSY1* was partially contributed the accumulation of more β-carotene and α-carotene. In addition, the transcriptional down-regulation of carotenoid degradation genes, like *OfNCED3* and *OfCCD4-1* was detected at least two flowering stages, which would help carotenoid accumulation under low temperature.

Among enzymes encoded by carotenoid degradation genes, OfCCD1 and OfCCD4 can cleave α-carotene, β-carotene for releasing α-ionone and β-ionone, which are two components of floral scent in sweet osmanthus [26]. In our previous study, we treated four sweet osmanthus cultivars by different temperatures [13]. Compared to 19 °C condition, the fully opening flowers of ‘Jingui Gui’ released lower relative content of α-ionone and β-ionone in 15 °C treatment [13], which was probably related to down-regulation of *OfCCD1-1* and *OfCCD1-2* at S2, and down-regulation of *OfCCD4-1* at S1 and S3. However, the lower relative content of α-ionone and β-ionone in 32 °C treatment than those in 19 °C treatment [13], was not consistent to the up-regulation of *OfCCD1-1*, *OfCCD1-2*, and *OfCCD4-1*. Therefore, other factors, such as water content and structures of petal cells which were probably involved in the emission of volatile compounds [26], may be also affected by relative high temperature.

Temperature is an important environmental signal, affecting plant growth, development and reproduction [27]. Temperature is also involved in the changes of coloration in plants by regulating the related genes directly or indirectly. Many researches are related to biosynthesis of another pigment group, anthocyanins. For instance, relative high temperature repressed pigmentation and anthocyanin biosynthetic genes in tuber flesh of potato (*Solanum tuberosum*), by reducing the transcription activators StAN1 and StbHLH1, and promoting expression of the transcription repressors like StMYB44 [28]. Relative low temperature promoted anthocyanin biosynthesis and fruit coloration of apple (*Malus domestica*), by inducing the transcription activator MdbHLH3 [29]. However, reports about the mechanism of regulation on carotenogenic genes by temperature are much fewer than those in anthocyanin biosynthesis. In our present research, as key carotenoid biosynthesis and degradation genes, the promoter activities of *OfLCYB1* and *OfCCD4-1* were directly affected by high temperature and low temperature (Fig. 6). According to *cis*-acting element analysis of *OfLCYB1* and *OfCCD4-1* promoters (Fig. 5), there are MYB binding sites and MYC binding sites which may also interact with some MYB transcription factors and MYC-type bHLH transcription factors, respectively. Besides, multiple *cis*-acting elements responding to phytohormones are probably related to abiotic stress, such as temperature variation [30, 31]. As reported, *cis*-acting elements like DRE and ABRE are involved in induction by low temperature [32, 33]. In Xiaohei poplar (*Populus simonii* × *Populus nigra*), PsnICE1 in response to cold stress can bind to ABRE element in promoters of stress-related genes for cold tolerance [34]. Previous studies have reported that OfWRKY3 and OfERF61 are two transcription factors activating the expression of *OfCCD4* [26, 35]. However, it remains to be further investigated whether any transcription factors of sweet osmanthus which are involved in regulation of carotenogenic genes by temperature exist.

## Conclusions

Our study revealed that high temperature (32 °C) greatly increased L* value, decreased a* value, and inhibited total carotenoid content and the content of α-carotene, β-carotene, α-cryptoxanthin, β-cryptoxanthin, lutein and zeaxanthin in petals of sweet osmanthus. By contrast, low temperature (15 °C) decreased L* value, increased a* value, and enhanced total carotenoid content and the content of individual carotenoids. The expression profiles of carotenoid biosynthesis and degradation genes showed that the response of carotenoid accumulation to different temperatures in sweet osmanthus was transcriptionally regulated. Among these genes, the promoter activities of *OfLCYB1* and *OfCCD4-1* were in response to different temperatures, suggesting that transcriptional regulation of these two genes probably plays a crucial role in temperature-regulated floral coloration of sweet osmanthus.

## Methods

### Plant materials and temperature treatments

Potted plants of sweet osmanthus ‘Jinqiu Gui’ with golden yellow flowers were grown in the resource nursery of Zhejiang Agriculture and Forestry University in Hangzhou, Zhejiang Province, China. Total nine individuals with similar height and crown size were selected for different temperature treatments. When the flowers developed at stage 0 (S0, the bud scales totally unfurled and the inside bracts just unfurled), three potted plants for each treatment were placed in plant growth incubators under a constant temperature (15 °C, 19 °C or 32 °C). The optimal and natural temperature for flower opening in sweet osmanthus is about 19 °C. Compared to the control (19 °C), 32 °C was employed as a high temperature treatment, while 15 °C was used as a low temperature treatment. For each temperature treatment, other culture conditions were the same as relative humidity of 80%, and a 12/12 h light/dark photoperiod with an illumination of 80 μmol•m^−2^•s^−1^. Petals from each plant under different treatments at S1, S2, and S3 were respectively sampled and immediately frozen in liquid nitrogen and stored at -80 °C for gene expression analysis. Besides, petals from each plant under different treatments at S3 were dried with silica gel at room condition for carotenoid content measurement.

### Floral color measurement

In addition to naked eye observation, floral color of each plant under different treatments during different developmental stages (S1-S3) was measured by an Minolta CR-10 portable colorimeter (Konica Minolta, Japan). Lightness (L*) and two chromatic components a* and b* of the CIEL*a*b* color coordinate were measured in day light conditions. Chroma (C*) was calculated based on the equation: C* = (a*^2^ + b*^2^)^1/2^ [36]. The floral color of each plant was determined on five replicates.

### Carotenoid content measurement

The extraction of total carotenoids was carried out following the method described by a previous study [37]. Briefly, the ground freeze petals were extracted with methanol firstly, and then hexane and a solution of NaCl (10%, w/v) were added. The mixture was continuously shaken until the plant material was colorless. Pooled organic phases were dried under nitrogen stream and saponified overnight using a 6% KOH methanolic solution. The carotenoids were subsequently reextracted with hexane: diethyl ether (3:1, v/v). The organic layers were combined and the solvent was removed under nitrogen stream.

Before HPLC analysis, the samples were dissolved in 2 ml MTBE (methyl *tert-butyl* ether) and filtered through a 0.22 μm micropore. The carotenoids were analyzed on a Shimadzu HPLC system (Kyoto, Japan), and separated on a C30 column (Shimadzu GL, Japan, 4.6 × 250 mm, 5 μm). Solvent A (methanol) and solvent B (MTBE) were used as the mobile phase at a flow rate of 0.8 ml/min and at 25 °C. Absorbance was detected at 450 nm. Identification and quantification of carotenoids was based on standards and our previous study [4].

### Quantitative real-time PCR (qRT-PCR) analysis

Total RNA was extracted from petals using RNAprep Pure Plant Kit (Tiangen, China). RNA quality and concentration were verified using 2100 Bioanalyzer RNA Nanochip (Agilent, Santa Clara, CA, USA). The first-strand cDNA was synthesized from 1 μg of DNA-free RNA with oligo(dT)_18_ primer using a PrimeScript® RT Reagent Kit (Takara, Japan).

qRT-PCR was performed using Roche Light Cycler 480II detection system with SYBR Premix Ex Taq (Takara, Dalian, China). The reaction mixture (20 μL total volume) contained 10 μL of SYBR Premix Ex Taq, 0.8 μL of each primer (10 μM), 2 μL of diluted cDNA, and 6.4 μL of ddH_2_O. Primers for 16 carotenogenic genes were obtained from our previous study [38]. The PCR program was carried out with an initial step of 95 °C for 30 s and 40 cycles of 95 °C for 5 s, 60 °C for 30 s; then 95 °C for 15 s, 60 °C for 1 min and 95 °C for 15 s for the dissociation stage. No-template controls for each primer set were included in every reaction, and the *OfACT* gene [39] was chosen as an internal control. The relative expression levels were calculated using the 2^−△Ct^ method [40]. Each analysis had three biological replicates.

### Cloning and *cis*-acting element analysis of *OfLCYB1* and *OfCCD4-1* promoters

Genomic DNA was extracted from leaves of sweet osmanthus ‘Jinqiu Gui’ using Plant Genomic DNA Kit (Tiangen, China). DNA quality and concentration were verified using 2100 Bioanalyzer RNA Nanochip (Agilent, Santa Clara, CA, USA). The promoter sequences of *OfLCYB1* and *OfCCD4-1* were cloned from the genomic DNA of sweet osmanthus ‘Jinqiu Gui’ according to *O. fragrans* genome database [41], and then analyzed for predicting *cis*-acting elements by using PlantCARE (http://bioinformatics.psb.ugent.be/webtools/plantcare/html/).

### Luciferase assays

Luciferase assays were performed to investigate the regulation on *OfLCYB1* and *OfCCD4-1* promoters by temperatures. The promoter sequences of *OfLCYB1* and *OfCCD4-1* were inserted into a pGreenII 0800-Luc vector, respectively. The construct of *OfLCYB1*pro::Luc or *OfCCD4-1*pro::Luc was transiently transformed into *N. benthamiana* leaves by using an Agrobacterium-mediated method. The transformed *N. benthamiana* plants were cultivated at 23 °C in darkness for 24 h, and then cultivated at 15 °C, 19 °C or 32 °C under a 12/12 h light/dark photoperiod with an illumination of 80 μmol•m^−2^•s^−1^ for 48 h. Then the transformed *N. benthamiana* leaves were examined by using a Tanon 5200 multi-imaging apparatus (Tanon, Shanghai, China) after being sprayed with D-luciferin sodium salt (Solarbio, Beijing, China). Each assay was performed with five biological replicates.

### Statistical analysis

One-way analysis of variance (ANOVA) was applied to the data using IBM SPSS Statistics 19.0 (IBM, Armonk, NY, United States). Duncan’s multiple range test was used to identify differences (*P* < 0.05) in floral color parameters, carotenoids content, the expression levels of carotenoid-related genes and luminescence intensity of promoters among the different temperature treatments.

## Supplementary Information

**Additional file 1: Fig. S1.** The HPLC chromatograms of carotenoids in petals of sweet osmanthus ‘Jinqiu Gui’ at full flowering stage with treatments of different temperatures.

## Data Availability

All data generated or analysed during this study are included in this published article and its additional file.
