# Supplementary material for: Temperature regulation of carotenoid accumulation in the petals of sweet osmanthus via modulating expression of carotenoid biosynthesis and degradation genes
Source: BMC Genomics. 2022 Jun 4;23:418. doi: 10.1186/s12864-022-08643-0 (PMC9166602; doi:10.1186/s12864-022-08643-0)
Supplement: Supplementary file 1 — Additional file 1: Fig. S1. The HPLC chromatograms of carotenoids in petals of sweet osmanthus ‘Jinqiu Gui’ at full flowering stage with treatments of different temperatures. [file 12864_2022_8643_MOESM1_ESM.pdf]

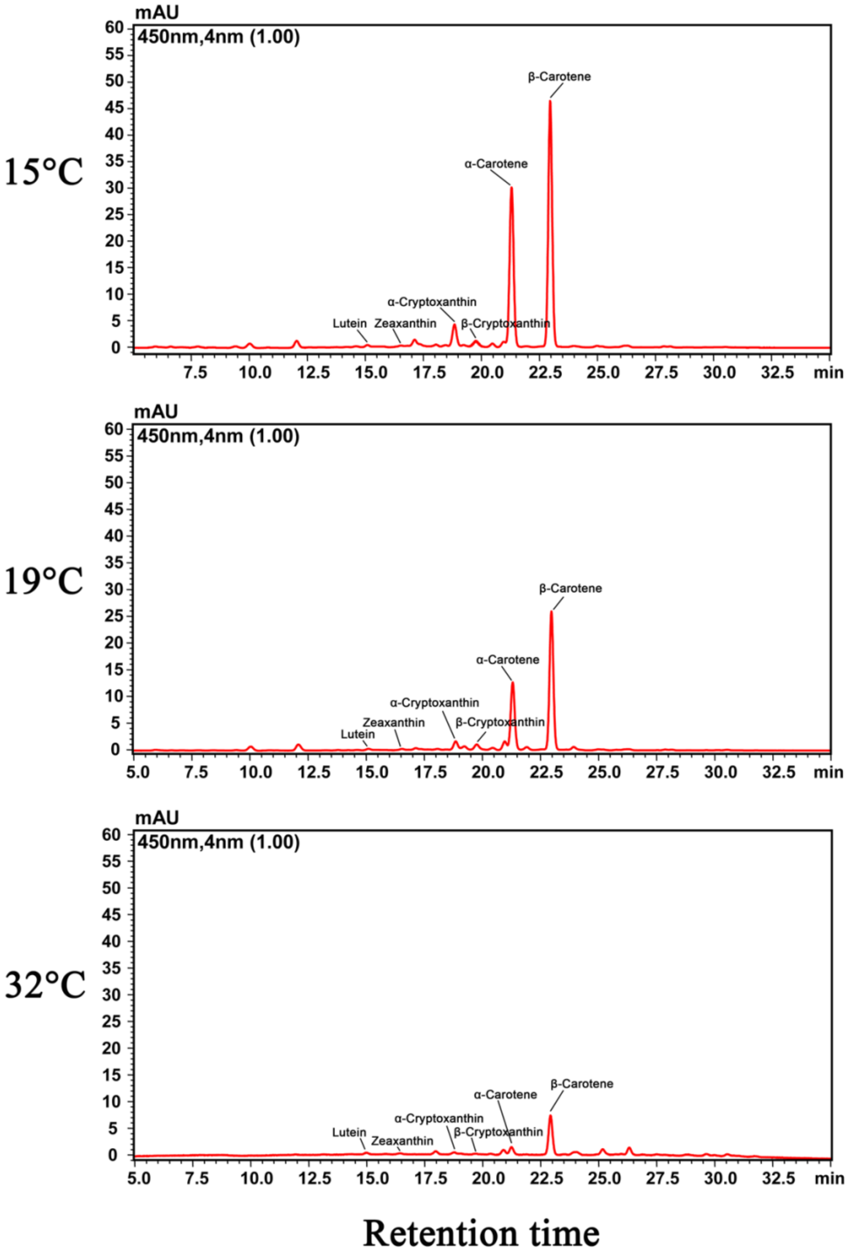

Fig. S1 The HPLC chromatograms of carotenoids in petals of sweet osmanthus 'Jinqiu Gui' at full flowering stage with treatments of different temperatures.
